# Advances in the Preparation, Structure and Bioactivity of Polysaccharides from *Lycium ruthenicum* Murr.: A Review

**DOI:** 10.3390/foods13131995

**Published:** 2024-06-25

**Authors:** Bing Liu, Jingyu Ma, Ting Li, Pei Li, Dehui Yan, Jun Zhu, Xinguo Zhang

**Affiliations:** 1 School of Life Science and Engineering, Lanzhou University of Technology, Lanzhou 730050, China; 17393302705@163.com (J.M.); tingli1097@163.com (T.L.); liliyll2018@163.com (P.L.); 18794206016@163.com (D.Y.); biodrug@163.com (X.Z.); 2 Gansu Institute of Standardization, Lanzhou 730000, China; zjjyl817@163.com

**Keywords:** *Lycium ruthenicum* Murr. polysaccharides, the preparation method, structural characteristics, bioactivity, mechanism

## Abstract

*Lycium ruthenicum* Murr. is rich in polysaccharides, and the polysaccharides in *Lycium ruthenicum* Murr. (LRPS) have various bioactivities, such as antioxidant activity, anti-tumor activity, neuroprotective activity, and immunomodulatory activity. It has broad prospects in the development of functional foods and pharmaceuticals. Researchers have found that the structural characteristics of LRPS, such as molecular weight, monosaccharide composition, primary structure, etc., have a significant impact on their bioactivities. Therefore, studying the structure of LRPS is of great significance in revealing their bioactivities and mechanisms. This study, based on introducing the preparation methods of LRPS, focuses on reviewing the research progress on the main structural characteristics, various bioactivities, and mechanisms of action of LRPS. In addition, the study provides prospects for the development of LRPS in the fields of food and medicine, aiming to provide theoretical support for its deep processing and application.

## 1. Introduction

**Figure 1:**
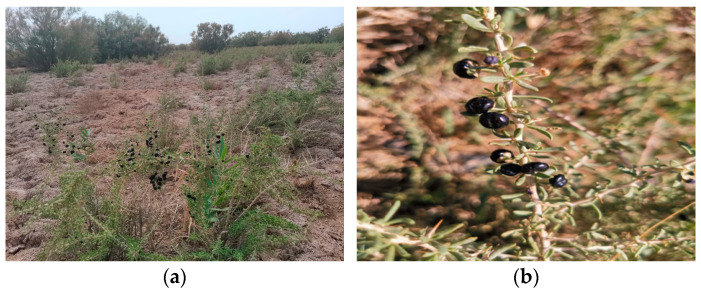
The images of *Lycium ruthenicum* plants (**a**) and fruits (**b**). (Taken in Guazhou County, Jiuquan City, Gansu Province in 2021).

*Lycium ruthenicum* Murr. is a plant belonging to the Solanaceae family and *Lycium* genus. The images of *Lycium ruthenicum* plants and fruits are shown in Figure 1. It is widely distributed in the arid deserts and saline-alkali areas of Northwest China, serving as an important halophytic medicinal and edible fruit tree. According to records in “Jing Zhu Ben Cao”, it is described as a slender, bushy shrub with many branches, gray bark, and purple-red fruits, which come in black and white varieties, commonly known as “gray pangma” and “black pangma”. It is mentioned to be effective in suppressing heat and treating gynecological diseases. Abundant morphological characteristics confirm the black variety as *Lycium ruthenicum* Murr., a validation also recognized in Tibetan medicine [1]. The berries contain high levels of nutrition and bioactive compounds, including polysaccharides, anthocyanins, amino acids, vitamins, minerals, betaine, and flavonoids [2,3,4,5]. Modern pharmacological studies have shown that *Lycium ruthenicum* Murr. exhibits various bioactivities, such as antioxidant activity [6], anti-fatigue activity [7], anti-tumor activity [8], anti-radiation activity [9], hypoglycemic activity [10], anti-osteoporosis [11], immunomodulatory [12], neuroprotection [13], hepatoprotective activity [14], prebiotic activity [15] and gastric mucosal protective effect [16].

From the year 2000 to 2024, a search was conducted in the China National Knowledge Infrastructure (CNKI) database and in the Science Direct database using “*Lycium ruthenicum* Murr. polysaccharides”. Approximately 206 related papers were found. The research mainly involves the extraction, isolation, structural characteristics, and biological activity studies of *Lycium ruthenicum* Murr. polysaccharides. Among these, 140 papers focus on the structure and biological activity of *Lycium ruthenicum* Murr., accounting for 67.96% of the total polysaccharides. Modern pharmacological studies indicate that polysaccharides play a crucial role as the important substance foundation for the diverse biological activities of *Lycium ruthenicum* Murr. Therefore, conducting fundamental theoretical research around *Lycium ruthenicum* Murr. polysaccharides is of significant importance. There is an urgent need for in-depth exploration and research on the active ingredients and the mechanisms of efficacy in *Lycium ruthenicum* Murr. polysaccharides.

Due to the diverse structures and bioactivities of *Lycium ruthenicum* Murr. polysaccharides, and the close relationship between them, this paper aims to systematically review the preparation methods, structural characteristics, bioactivities, and mechanisms of action of *Lycium ruthenicum* Murr. polysaccharides. The goal is to provide a comprehensive overview that can serve as a reference for the in-depth processing and further commercial application of *Lycium ruthenicum* Murr. polysaccharides.

## 2. Extraction and Purification

### 2.1. Extraction of Lycium ruthenicum Murr. Polysaccharides

Polysaccharides, as crucial active components in *Lycium ruthenicum* Murr., have garnered increasing attention and research from scholars. The effective extraction of polysaccharides is a prerequisite for conducting studies on structural characteristics, bioactivity evaluation, and mechanisms, serving as the foundation for the development of polysaccharides in functional foods and pharmaceuticals.

**Figure 2:**
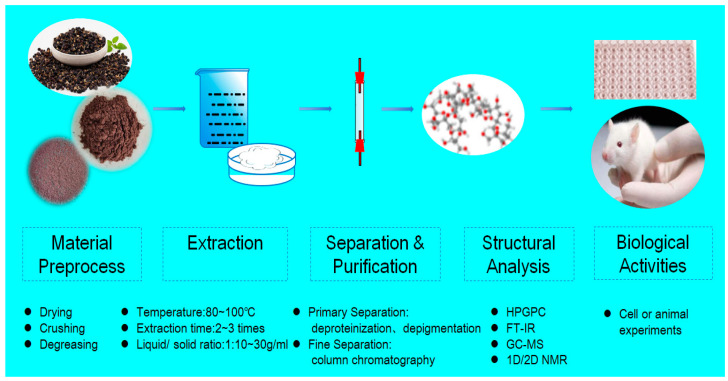
Flow chart of *Lycium ruthenicum* Murr. polysaccharide extraction.

Before extracting polysaccharides from *Lycium ruthenicum* Murr., the fruits need to undergo processes such as impurity removal, drying, crushing, and defatting. The most common method for extracting polysaccharides from *Lycium ruthenicum* Murr. is hot water extraction. This method takes advantage of the property that polysaccharides dissolve in water but not in ethanol. The main factors affecting the efficiency of polysaccharide extraction include extraction temperature, material ratio, and extraction time. In optimizing the extraction conditions of *Lycium ruthenicum* Murr. polysaccharides, it has been found that the extraction temperature significantly influences the polysaccharide yield, while extraction time and material ratio have less pronounced effects, with the latter having the smallest impact on polysaccharide yield [11]. This suggests that the extraction temperature is the most significant factor in polysaccharide extraction. Research by Shen Jialin [17] revealed that at the extraction temperature of 90 °C, the yield of *Lycium ruthenicum* Murr. polysaccharides reached its highest point. Beyond this temperature, the yield began to decrease. This indicates that within a certain temperature range, the yield of *Lycium ruthenicum* Murr. polysaccharides increases with the temperature. Higher extraction temperatures contribute to enhanced molecular thermal motion, making polysaccharides more soluble. However, it is important to note that *Lycium ruthenicum* Murr. polysaccharides are unstable at high temperatures and may undergo degradation, leading to a decrease in polysaccharide yield. The flow chart of *Lycium ruthenicum* Murr. polysaccharide extraction is given in Figure 2.

**Figure 3:**
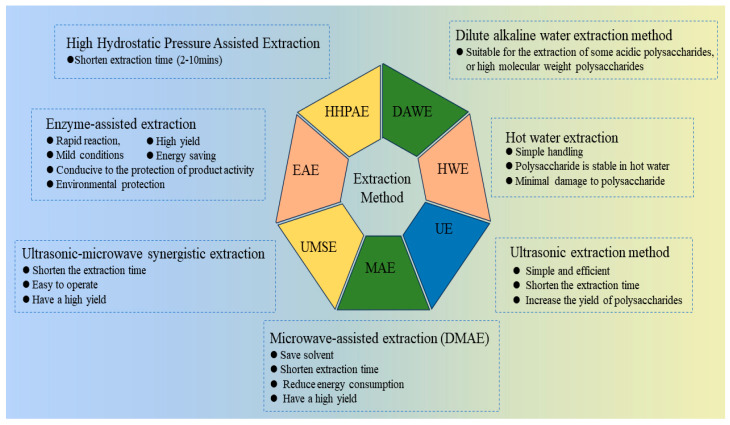
Extraction methods related to *Lycium ruthenicum* Murr. polysaccharide comparisons in terms of advantages. (HWE: Hot water extraction; UEM: Ultrasonic extraction method; MAE: Microwave-assisted extraction; WMSE: Ultrasonic–microwave synergistic extraction; EAE: Enzyme-assisted extraction; HHPAE: High Hydrostatic Pressure Assisted Extraction; DAWE: Dilute alkaline water extraction method).

In recent years, several auxiliary extraction methods have been employed to enhance the yield of polysaccharides extracted from *Lycium ruthenicum* Murr. using hot water. The main methods include ultrasound-assisted extraction, microwave-assisted extraction, ultrasound–microwave synergistic extraction, and enzyme-assisted extraction. These approaches can increase the yield of LRPS through different pathways, but they also have their respective drawbacks. In the future, supercritical fluid extraction may also be applied to the extraction of polysaccharides from *Lycium ruthenicum* Murr.. The theories and advantages/disadvantages of various extraction methods are summarized in Table 1 and Figure 3.

### 2.2. Purification of LRPS

Crude LRPS may carry some associated proteins, pigments, monosaccharides, and other impurities. Further purification is required, through methods such as deproteinization, decolorization, dialysis, ethanol precipitation. Afterward, chromatographic techniques involving ion exchange columns and gel filtration columns are employed for additional separation. Chromatography is a technique that utilizes the different distribution ratio of substances between the stationary phase and the mobile phase to achieve separation [28]. Commonly used ion exchange resins for the separation of polysaccharides and purification include DEAE-Cellulose, DEAE-Sepharose Fast Flow and others, such as Sephadex, Sepharose, Sephacryl, Bio-gel, etc. [29]. It is worth noting that, due to the high cost and small scale of column chromatography, there have been no reported methods suitable for industrial-scale purification of LRPS.

## 3. Structural Characteristics

Polysaccharides, also known as glycans, are naturally occurring carbohydrate polymers composed of numerous monosaccharide units linked by glycosidic bonds. They typically have molecular weights ranging from tens of thousands to even millions of Da [30]. The structure characteristics of LRPS include their molecular weight, monosaccharide composition, primary structure, and higher-order structure. The primary structure encompasses sugar unit composition and arrangement, the linkage pattern of adjacent sugar units, sugar chain branching, substitution group positions, anomeric configurations, etc. The primary structure serves as the material basis for the formation of higher-order structures and the manifestation of polysaccharide efficacy. Currently, various modern analytical techniques can be employed to elucidate the structural features of LRPS. These techniques include High-Performance Liquid Chromatography (HPLC), Fourier Transform Infrared Spectrometer (FT-IR), Gas Chromatography–Mass Spectrometry (GC-MS), one-dimensional and two-dimensional Nuclear Magnetic Resonance analyses (NMR), acid hydrolysis, periodate oxidation, methylation analysis, etc. [31]. Table 2 summarizes the molecular weight, monosaccharide composition, and glycosidic linkages of the different kinds of LRPS.

### 3.1. The Molecular Weight of LRPS

Polysaccharides are naturally occurring carbohydrate polymers, with molecular weights typically ranging from tens of thousands to even millions of Da [32]. The detection of the molecular weights of polysaccharides, such as those found in *Lycium ruthenicum* Murr., is commonly performed using High-Performance Gel Permeation Chromatography combined with a refractive index detector. Molecular weight is considered a crucial factor in characterizing the natural polysaccharide structure. The molecular weight distribution of LRPS is quite broad, typically falling within the range of 17.0 to 2650 kDa, as illustrated in Table 2. Variations in the molecular weight of LRPS depend on the specific types of polysaccharides present. For instance, Peng [33,34] isolated and purified two different polysaccharides, LRGP1 and LRGP3, from the same source of *Lycium ruthenicum* Murr., with molecular weights of 5.62 × 10^4^ and 7.56 × 10^4^ Da, respectively.

### 3.2. The Monosaccharide Composition of LRPS

**Figure 4:**
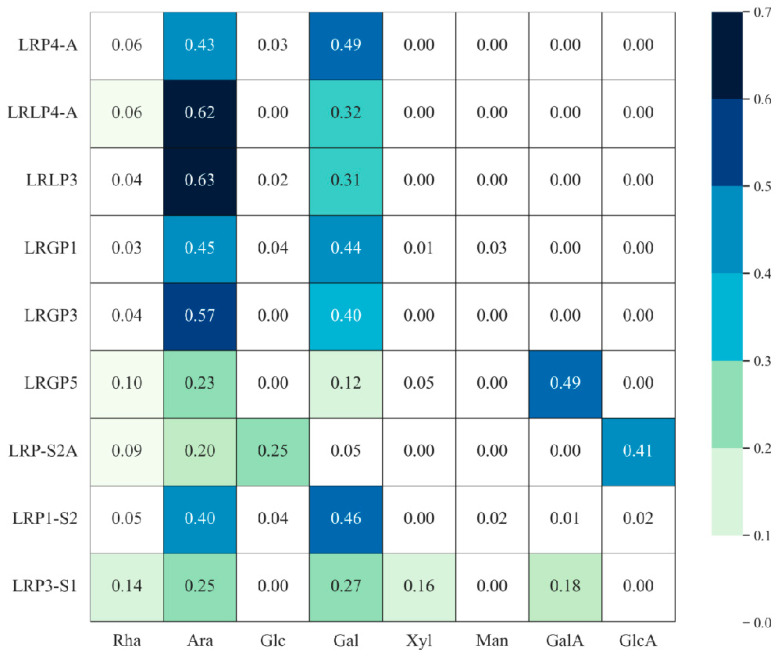
Heat map of monosaccharide composition of LRPS based on literature research.

The monosaccharide composition of LRPS includes the types of monosaccharide present and the proportion of these monosaccharides in the overall composition. LRPS is primarily composed of rhamnose (Rha), arabinose (Ara) and galactose (Gal), with some containing glucose (Glc), xylose (Xyl) and mannose (Man) and small amounts of glucuronic acid (GlcA) and galacturonic acid (GalA). As shown in Table 2, different components of LRPS exhibit variations in monosaccharide composition. For example, Lv et al. [30] applied the method of water extraction and ethanol precipitation to obtain crude polysaccharides (LRP) from *Lycium ruthenicum* Murr.. After purification through anion exchange chromatography and gel column chromatography, they obtained a polysaccharide fraction, LRP4-A, with the monosaccharide composition mainly consisting of Rha, Ara, Glu, and Gal, in a molar ratio of 1:7.6:0.5:8.6. Peng [35] extracted and isolated LRPG5 from *Lycium ruthenicum* Murr.. A monosaccharide composition analysis indicated that LRPG5 is mainly composed of Rha, Ara, Xyl, Gal, and GalA, with a molar ratio of 1.0:2.2:0.5:1.2:4.7. The heat map analysis of the monosaccharide composition of various reported LRPS is shown in Figure 4.

### 3.3. The Primary Structure of LRPS

**Figure 5:**
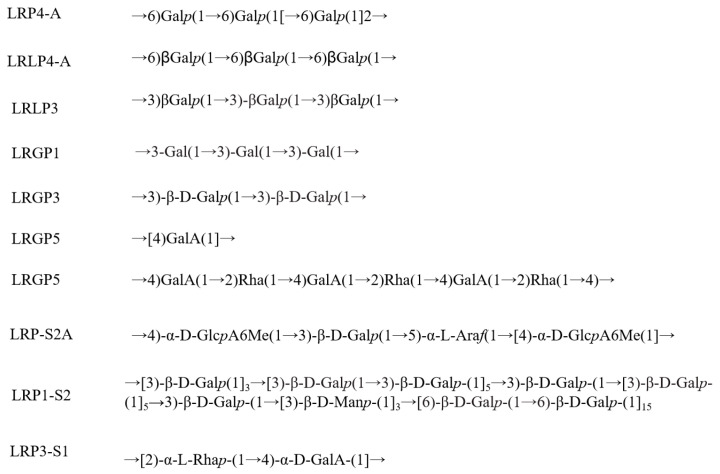
The main repeated units in the structures from Table 2 of LRP4-A, LRLP4-A, LRLP3, LRGP1, LRGP3, LRGP5, LRP-S2A, LRP1-S2, and LRP3-S1.

Research on the primary structure of LRPS mainly focuses on the linkage patterns between monosaccharides, branches, and methylation patterns. As shown in Table 2, different polysaccharide components exhibit differences in their overall structure due to variations in methylation substitution positions, methylation patterns, or the proportion between different methylation patterns. For instance, Wang et al. [11] discovered that the polysaccharide LRP-S2A, extracted from *Lycium ruthenicum* Murr., consists of a main chain in its primary structure composed of α-(1→4)-D-GalpA, α-(1→4)-2-O-acetyl-D- GalAMe, α-(1→2,4)-L-Rha*p* and β-(1→4)-D-Glc*p* repeat units, while the side chain includes β-(1→3)-D-Gal*p*, α-(1→4)-D-Gal*p*A, α-(1→3,5)-L-Ara*f* and α-1-L-Ara*f*. He et al. [36] extracted a polysaccharide from *Lycium ruthenicum* Murr. named LRP1-S2, whose main chain of its primary structure is composed of β-(1→3)-D-Gal*p*, β-(1→6)-D-Gal*p*, β-(1→3,6)-D-Gal*p* and β-(1→3)-D-Man*p*, while the side chain includes T-β-D-Gal*p*, T-α/β-L-Ara*f*, α-(1→5)-L-Ara*f*, T-α-L–Rha*p*, α-(1→2)-L-Rh*a*, β-(1→4)-D-Glc*p*, T-β-D-GlcA, β-(1→4)-D-GlcA and α-(1→4)-D-GalA. The main domains of the LRPS are shown in Figure 5.

**Table 2:** The summary of structural features of *Lycium ruthenicum* Murr. polysaccharides.

Number	Designation of Polysaccharides	Separation and Purification Methods	Molecular Weight (kDa)	Monosaccharide Composition	Glycosidic Linkage Pattern	Branch Point	References
1	LRP4-A	DEAE-Cellulose-52 (0.5 M NaHCO_3_) Sephadex G-100 (0.1 M NaCl)	105	Rha:Ara:Glc:Gal =1:7.6:0.5:8.6	→6)-Gal*p*-(1→	O-3 of Gal	[30]
2	LRLP4-A	DEAE-Cellulose-52 (0.5 M NaHCO_3_) Sephadex G-100 (0.1 M NaCl)	135	Rha:Ara:Gal =1:10.3:5.3	→6)-β-Gal*p*-(1→	O-3 of Ara or Gal	[37]
3	LRLP3	DEAE-Cellulose-52 (0.1 M NaHCO_3_) Sephadex G-100 (0.1 M NaCl)	79.4	Ara:Gal:Rha:Glc =2:1:0.12:0.06	→3)-β-Gal*p*-(1→	/	[38]
4	LRGP1	DEAE-Cellulose-52 (0.05 M NaHCO_3_) Sephadex G-100 (0.1 M NaCl)	56.2	Rha:Ara:Xyl:Man:Glc:Gal =0.65:10.71:0.33:0.67:1:10.41	→3)-Gal*p*-(1→	O-6 of Ara and Gal	[33]
5	LRGP3	DEAE-Cellulose-52 (0.15 M NaHCO_3_) Sephadex G-100 (0.1 M NaCl)	75.6	Rha:Ara:Gal =1.0:14.9:10.4	→3)-β-D-Gal*p*-(1→	O-6 of Gal or Ara	[12,34]
6	LRGP5	DEAE-Cellulose-52 (0.5 M NaHCO_3_) Sephadex G-100 (0.1 M NaCl)	137	Rha:Ara:Xyl:Gal:GalA =1.0:2.2:0.5:1.2:4.7	→4)-GalA-(1→ →1)-Rha-(→2	O-4 of Rha	[35]
7	LRP-S2A	DEAE Sepharose Fast Flow Sephacryl S-500 HR	2650	Rha:Ara:Gal:Glc:GlcA =1.00:2.07:0.57:2.59:4.33	→4)-6-O-α-D-Glc*p*A-(1→ →4)-2-O-acetyl-α-D-Glc*p*-(1→ →2,4)-α-L-Rha*p*-(1→ →4)-β-D-Glc*p*A-(1→	/	[11]
8	LRP1-S2	DEAE Sepharose Fast Flow Sephacryl S-100 HR	17.0	Gal:Ara:Rha:Glc:GlcA: Man:GalA =46.2:40.2:5.1:4.0:2.3:1.7:0.5	→3)-β-D-Gal*p*-(1→ →6)-β-D-Gal*p*-(1→ →3,6)-β-D-Gal*p*-(1→ →3)-β-D-Man*p*-(1→	/	[36]
9	LRP3-S1	DEAE Sepharose Fast Flow (0.2 M NaCl) Sephacryl S-300 HR (0.2 M NaCl)	114.8	Rha:GalA:Gal:Xyl:Ara =14.4:17.7:26.6:16.4:24.9	→2)-α-L-Rha*p*-(1→ →4)-α-D-GalA-(1→	/	[39]

## 4. Bioactivities

Polysaccharides, as one of the primary active components in *Lycium ruthenicum* Murr., have been a focal point of research, particularly regarding their biological activities. With the advancement of modern pharmacological studies, various biological activities of LRPS have been extensively investigated. Research indicates that LRPS show diverse biological activities, including antioxidant activity [6], anti-fatigue activity [7], anti-tumor activity [8], anti-radiation activity [9], hypoglycemic activity [10], anti-osteoporosis [11], immunomodulatory [12], neuroprotection [13], hepatoprotective activity [14], prebiotic activity [15] and a gastric mucosal protective effect [16].

### 4.1. Immunomodulatory Activity

**Figure 6:**
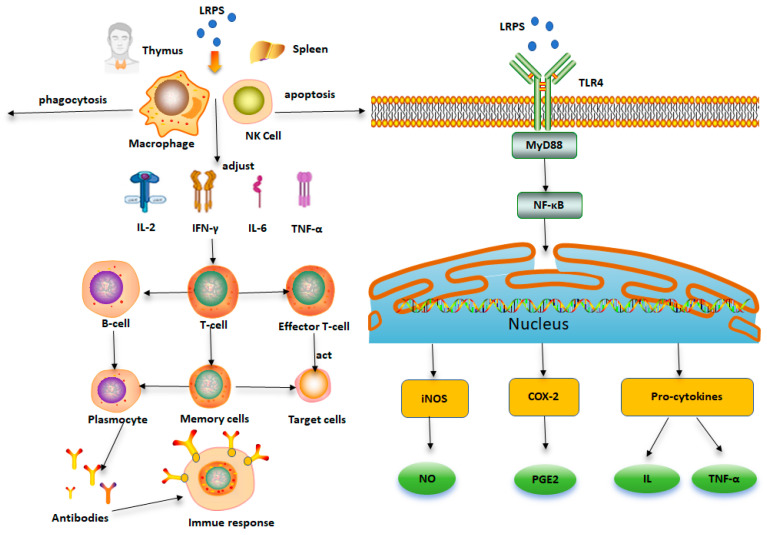
The potential immunomodulatory mechanisms of LRPS.

The immune response serves as a crucial defense mechanism in the body against external threats, recognizing and defending against the harm caused by foreign pathogens and toxins. Natural polysaccharides are considered effective immune modulators, and modern pharmacological research indicates that LRPS possess immunomodulatory activity. Researchers have delved into the immunomodulatory effects and mechanisms of polysaccharides at the tissue and organ level, cellular level, molecular level, and intestinal microbiota level. The potential immunomodulatory mechanisms of LRPS are shown in Figure 6. The studies demonstrate that the immunomodulatory effects of polysaccharides are not limited to a single aspect but involve multi-stage, multi-target regulation of the body’s immune functions [40]. Polysaccharides, when taken orally, are resistant to digestion by enzymes in saliva or gastric fluids and are primarily absorbed into the bloodstream through the intestines. By recognizing and binding to specific receptors on the surface of immune cells, polysaccharides activate downstream signaling pathways within the cells, promoting the expression of relevant cytokines. This, in turn, initiates the immune response and exerts immunomodulatory effects. The identified polysaccharide receptors include Toll-like receptors (TLRs), scavenger receptors (SR), complement receptor 3 (CR3), Dectin-1, cluster of differentiation 14 (CD14), and mannose receptor (MR) [41]. The activation of these pattern recognition receptors leads to signal transduction through pathways such as mitogen-activated protein kinases (MAPKs) and nuclear factor-κB (NF-κB). Subsequently, a series of kinase-regulated phosphorylation reactions occur, activating the transcription of various immune-related genes, thereby exerting immunomodulatory effects. LRPS have been found to contain abundant arabinogalactan. Studies suggest that the sulfate content of arabinogalactan is related to its ability to activate macrophages. The arabinogalactan backbone is essential for complement activity expression, and the arabinose side chain can activate macrophages [42]. Peng et al. have demonstrated that LRPS can alleviate inflammation induced by lipopolysaccharide (LPS) by inhibiting the TLR4/NF-κB signaling pathway. These studies collectively highlight the immunomodulatory activity of LRPS [43].

### 4.2. Antioxidant Activity

During the normal process of oxidation and reduction in cellular metabolism, the body’s cells produce a certain amount of reactive oxygen species (ROS). Simultaneously, the body possesses a series of antioxidant substances to maintain the balance of the oxidative–antioxidative system. Examples include superoxide dismutase (SOD), catalase, glutathione reductase, and antioxidants such as ubiquinol and vitamins [44]. When the body is exposed to harmful conditions, an excess production of reactive oxygen species (ROS) occurs, leading to the imbalance between oxidation and antioxidation, known as oxidative stress (OS). Following oxidative stress, the body generates various toxic metabolic byproducts, such as malondialdehyde (MDA), a final product of lipid peroxidation. MDA can cause the cross-linking polymerization of life macromolecules such as proteins and nucleic acids, leading to cellular toxicity. This can result in DNA oxidative damage, inflammatory infiltration of neutrophils, abnormal protein expression, and the development of various diseases. These diseases may include fatigue, cardiovascular and cerebrovascular diseases, atherosclerosis, Parkinson’s disease, Alzheimer’s disease, type II diabetes, cataracts, liver damage, pulmonary fibrosis, osteoporosis, and cancer [45]. Therefore, mitigating and preventing the occurrence of various diseases can be achieved through antioxidant mechanisms.

Liu et al. [46] employed the method of dynamic microwave-assisted extraction to extract polysaccharides from *Lycium ruthenicum* Murr. and explored their antioxidant activity through in vitro experiments. The study revealed that the mechanism of the antioxidant action of polysaccharides from *Lycium ruthenicum* Murr. includes the scavenging of 1,1-diphenyl-2-trinitrophenylhydrazine (DPPH) free radicals, hydrogen peroxide, superoxide anion radicals [24,47], and the activity against ABTS+ [48]. The polysaccharides demonstrated the strong antioxidant activity, which was associated with the molecular weight, the glycuronic acid content, and the monosaccharide composition [49]. Liu Yang et al. [37] found that the polysaccharide LRLP3 from *Lycium ruthenicum* Murr. leaves effectively inhibited protein oxidation damage induced by Cu^2+^/H_2_O_2_ and oxidative damage to cells induced by H_2_O_2_, with an increasing antioxidant capacity with higher concentrations. LRLP3 also exhibited a promoting effect on the proliferation of mouse spleen cells induced by non-induced, concanavalin A (ConA)-induced, or lipopolysaccharide (LPS)-induced conditions. Furthermore, Duding et al. [50] discovered that purified polysaccharides from *Lycium ruthenicum* Murr. exhibited stronger antioxidant ability compared to crude polysaccharides, and the antioxidant capacity increased gradually with concentration. Xu et al. [51] found that polysaccharides from *Lycium ruthenicum* Murr. in Minqin, Gansu, successfully improved the antioxidant capacity of Streptococcus thermophilus G2 and Lactobacillus paracasei L9. Tan Yangyang et al. [6] found that polysaccharides from *Lycium ruthenicum* Murr. enhanced the antioxidant capacity of ARPE-19 cells induced by H_2_O_2_, and possibly inhibited ARPE-19 cell pyroptosis by downregulating the protein expression of NLRP3, Caspase-1, and IL-1β. Moreover, the purified polysaccharide LMP-2-1 exhibited higher activity than crude polysaccharides and could be considered a potential therapeutic drug for age-related macular degeneration (AMD). Chen et al. [52] determined the oxygen radical absorbance capacity (ORAC) of *Lycium ruthenicum* Murr. as 587 μmoleTE/g. Cui et al. [53] found that polysaccharides from *Lycium ruthenicum* Murr. effectively alleviated the imbalance in the internal environment caused by excessive training. They suppressed the overexpression of proteins in the MAPK signaling pathway, activated the MAPK signaling transduction system in the skeletal muscles of rats induced by excessive training, induced the expression of antioxidant enzyme genes, enhanced the oxygen free radical scavenging capacity of the body, especially in skeletal muscles, and regulated the body’s ability to resist oxidative stress damage. This helped prevent and delay the occurrence and development of oxidative stress damage and exercise fatigue.

### 4.3. Anti-Osteoporosis Activity

Osteoporosis is a systemic metabolic bone disease characterized by an imbalance between osteoclast bone resorption and osteoblast bone formation. It is primarily characterized by microarchitectural deterioration, low bone mass, and an increased risk of fractures. Osteoporosis is commonly observed in the elderly population and postmenopausal women [54]. Research suggests that enhancing osteoblast differentiation is beneficial in preventing osteoporosis. Wang et al. [11] extracted polysaccharides from *Lycium ruthenicum* Murr., and different components of these polysaccharides were found to promote osteoblast proliferation to varying degrees. Additionally, all polysaccharide components significantly increased the alkaline phosphatase (ALP) activity of osteoblast cells. Among them, the polysaccharide component named LRPS2A exhibited the most significant effects, indicating its potential as a homogeneous polysaccharide with the ability to promote osteoblast differentiation.

### 4.4. Neuroprotective Activity

**Figure 7:**
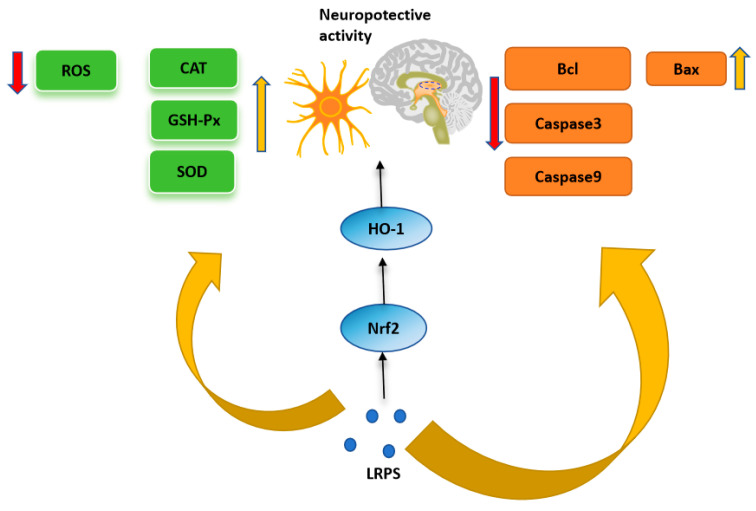
The potential neuroprotective mechanisms of LRPS.

Neurodegenerative diseases are a class of disorders characterized by functional impairment, due to factors such as the degeneration of neurons and their myelin sheaths. These diseases, primarily characterized by cognitive and motor dysfunction, are prevalent in the elderly population, leading to a high disability and mortality rate. Common specific diseases include Alzheimer’s disease (AD), Parkinson’s disease (PD), Huntington’s disease (HD), and Amyotrophic Lateral Sclerosis (ALS) [55]. Numerous studies suggest that polysaccharides exert protective effects on the central nervous system through various mechanisms. The mechanisms underlying their neuroprotective activity include antioxidant activity, the modulation of signaling pathways such as Nrf2/HO-1, NF-κB, PI3K/Akt, MAPKs, the promotion of neurite growth in neuronal cells, and support for neuronal nutrition [56]. The potential neuroprotective mechanisms of LRPS are shown in Figure 7. In a study by Deng et al. [13], the impact of LRP3 on embryonic day 14–16 Sprague Dawley rat primary cortical neurons subjected to oxygen-glucose deprivation/reoxygenation (OGD/R) was investigated. The results showed that LRP3 could alleviate the increase in reactive oxygen species (ROS) generation and enhance the activities of catalase (CAT), superoxide dismutase (SOD), and glutathione peroxidase (GPx) induced by OGD/R. Furthermore, LRP3 treatment significantly reduced caspase-3 activity in the cortical neurons induced by OGD/R. LRP3 pretreatment also mitigated the OGD/R-induced increase in Bax expression and decrease in Bcl-2 expression. Additionally, LRP3 significantly induced the expression of nuclear factor-E2-related factor 2/hemeoxygenase-1 (Nrf_2_/HO-1) in cortical neurons induced by OGD/R, indicating that LRP3 significantly enhanced the cell viability of cortical neurons induced by OGD/R and had a neuroprotective effect against injury in rat primary cortical neurons induced by OGD/R.

### 4.5. Anti-Tumor Activity

**Figure 8:**
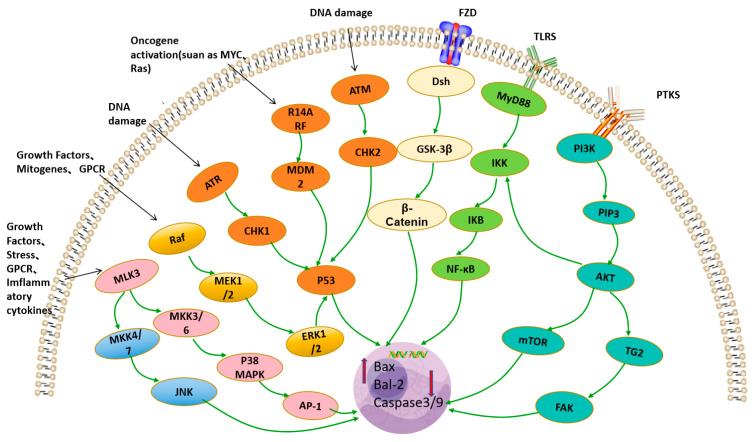
The potential anti-tumor mechanisms of LRPS.

Cancer is a disease characterized by abnormal cell proliferation due to various factors such as genetics, the environment, and current lifestyle factors. Traditional cancer treatment drugs often have significant side effects on the body. Recent studies suggest that polysaccharides exhibit certain anti-tumor activities. The potential anti-tumor mechanisms of LRPS are shown in Figure 8. Zhang et al. [39] prepared LRP3-S1, a polysaccharide from *Lycium ruthenicum* Murr., and found that it had a dose-dependent inhibitory effect on the proliferation of pancreatic cancer cells (BxPC-3, PANC-1, and AsPC-1), with minimal cytotoxicity to normal cells (HPDE6-C7 and LO2). The mechanism of cancer cell inhibition was associated with blocking the MAPK and FAK/AKT/GSK-3β signaling pathways. Another polysaccharide component, LRP1-S2, from *Lycium ruthenicum* Murr., was found to inhibit pancreatic cancer cell proliferation both in vitro and in vivo, with no significant cytotoxicity to normal pancreatic (HPDE6-C7) and liver (LO2) cells. Mechanistic studies suggest that it may induce apoptosis in BxPC-3 cells by deactivating the P38 MAPK/NF-κB and GSK-3β/β-Catenin signaling pathways [36]. These findings indicate that LRP3-S1 and LRP1-S2 may serve as potential lead compounds for anti-tumor agents, with the potential to be developed as novel inhibitors for pancreatic ductal adenocarcinoma, suitable for the development of functional foods and new drugs.

### 4.6. Anti-Radiation Activity

**Figure 9:**
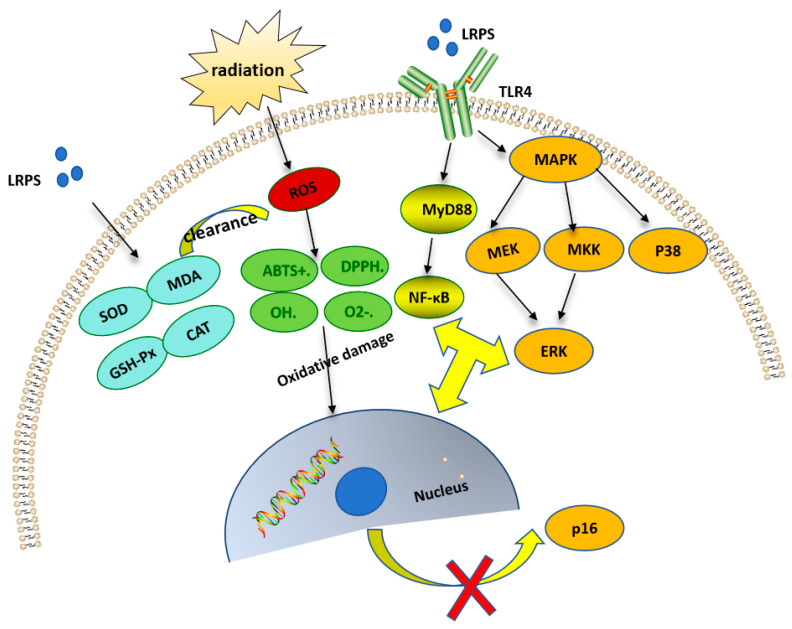
The potential Anti-radiation mechanisms of LRPS.

Radiation refers to the phenomenon where a portion of electromagnetic energy emitted by a radiation source (such as electromagnetic waves) departs from the source and propagates to a distance without returning. This energy can take the form of electromagnetic waves or particles (such as α particles, β particles, etc.) and includes ultraviolet radiation, thermal radiation, microwaves, radio waves, cosmic rays, X-rays, etc. Small amounts of radiation exposure do not pose a threat to human health, but excessive exposure to radiation can harm the body, potentially leading to diseases or even death. Research indicates that the anti-radiation mechanisms (Figure 9) of polysaccharides are mainly achieved through improving oxidative damage, regulating the immune system, modulating cell apoptosis, and protecting the DNA and hematopoietic system [57]. Based on the current literature, the immune modulation of polysaccharides against radiation is primarily mediated through three signaling pathways: the MAPK signaling pathway, the PI3K/Akt signaling pathway, and the signaling pathway mediated by membrane immunoglobulin (mIg) complex receptors [58]. Wang et al. [9] found that HaCaT cells, after 1 h of exposure to 30 mJ/cm^2^ UVB, experienced oxidative stress, produced a large amount of ROS, triggered the DNA damage response mechanism, led to cell cycle arrest, inhibited cell proliferation, exhibited blurred cell morphology, and showed signs of death floating. However, when HaCaT cells were treated with 2 g/L solution of *Lycium ruthenicum* Murr. polysaccharides for 6 h before 30 mJ/cm^2^ UVB exposure, the cells were able to recover their phenotype, enhance cell proliferation activity, and significantly reduce MDA and TNF-α levels. From this, it was concluded that LRPS may clear accumulated ROS by inhibiting oxidative stress reactions and simultaneously blocking the p38MAPKs signaling pathway, downregulating p16 protein expression and thereby alleviating photo aging. Duan Yabin [59] found that LRPS could promote the recovery of body weight, blood profile, bone marrow DNA, thymus, and spleen in mice after radiation exposure. They enhanced antioxidant enzyme activity, reduced the expression of caspase-3, caspase-6, and p53 proteins, and decreased cell apoptosis.

### 4.7. Anti-Fatigue Activity

Exercise-induced fatigue is a sub-healthy state where the body’s exercise function decreases due to the complex interplay of multiple factors. Usually, pathological fatigue, normally occurring along with chronic diseases, should be cured properly. Recent research indicates that dietary supplementation with herbs or natural substances from food sources can effectively delay exercise-induced fatigue. The underlying mechanisms include increasing the body’s energy substrate reserves, promoting fat utilization for energy, maintaining redox homeostasis, enhancing mitochondrial biogenesis, repairing mitochondrial damage, reducing metabolite accumulation, and inhibiting the accumulation of neurotransmitters [60]. Wang et al. [7], through experiments involving weighted swimming, confirmed the anti-fatigue effect of LRPS on normal mice. LRPS extended the swimming time of normal mice, alleviated oxidative stress, lowered post-exercise serum urea and blood lactate levels, increased the reserves of liver glycogen and muscle glycogen, and improved the exercise load capacity of normal mice, making them less prone to fatigue. Ni et al. [61], through chromatographic separation, obtained the purified polysaccharide LRWP from *Lycium ruthenicum* Murr. and found that LRWP could inhibit lipid oxidation, modify the activities of enzymes, such as glutathione peroxidase (GPx), superoxide dismutase (SOD), lactate dehydrogenase (LDH), and creatine kinase (CK), mobilize triglycerides during exercise, protect cell membranes, and reduce the immobility time of mice in weighted swimming experiments.

### 4.8. Hypoglycemic Activity

Diabetes mellitus (DM) is a disease characterized by high blood sugar levels due to pancreatic dysfunction, leading to reduced insulin secretion, abnormal insulin action, or a combination of both. It can be classified into Type 1 diabetes (T1DM) and Type 2 diabetes (T2DM). Typical symptoms include “polydipsia” (excessive thirst), “polyuria” (excessive urination), “polyphagia” (excessive hunger), and weight loss [62]. Both in vivo and in vitro experiments have shown that polysaccharides have effects on lowering blood sugar, reducing blood lipids, exhibiting antioxidant and anti-inflammatory properties, enhancing pancreatic β-cell mass, and alleviating β-cell dysfunction [63]. They achieve this by enhancing the insulin signaling pathway through the insulin receptor, activating the PI3K/Akt pathway, and ultimately regulating the ERK/JNK/MAPK pathway [64]. In an animal experiment conducted by Wang et al. [7], using a preventive pre-treatment and post-modeling approach and comparing with metformin hydrochloride as a control, although LRPS had a weaker hypoglycemic effect compared to metformin hydrochloride, it significantly lowered blood glucose levels in diabetic mice, increased the activity of serum and hepatic superoxide dismutase (SOD), reduced the content of serum and hepatic malondialdehyde (MDA), and promoted the conversion of glucose into hepatic glycogen.

### 4.9. Hepatoprotective Activity

**Figure 10:**
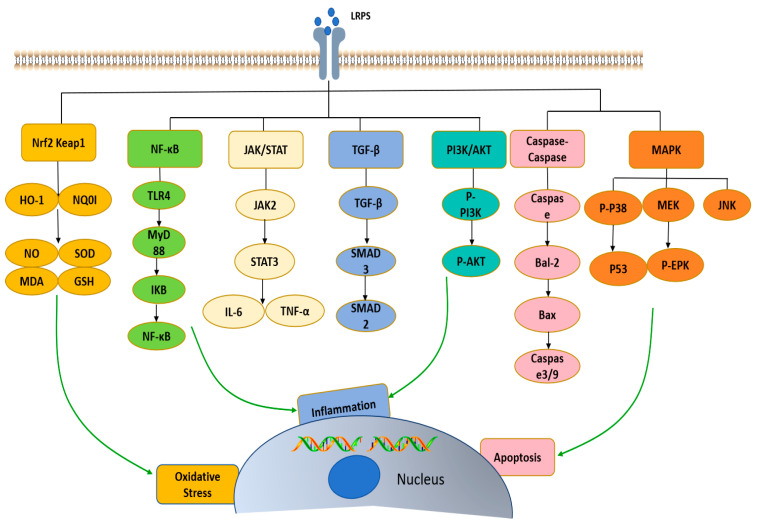
The potential hepatoprotective mechanisms of LRPS.

The liver is a crucial organ for maintaining physiological activities in the human body. It effectively breaks down and metabolizes substances entering the body, particularly toxic substances. It plays an irreplaceable role in maintaining metabolic homeostasis and immunity [65]. Liver damage is a common pathological process that can be caused by various factors, including viral infections, alcohol, drug abuse, biological and chemical toxins, and autoimmune attacks on liver cells. Liver damage can lead to conditions such as fatty liver, cirrhosis, fibrosis, and may even trigger cancer. Qu et al. [66] reviewed the hepatoprotective effects of plant polysaccharides through the pathological process of inflammation, apoptosis and oxidative stress by regulating NF-κB, JAK/STAT, TGF-β, PI3K/AKT, MAPK, caspase cascade, p53 and Nrf2-Keap1 pathways, and lipid metabolism, as well as the effect of cytochrome P450 enzymes. The potential hepatoprotective mechanisms of LRPS are shown in Figure 10. Guo et al. [67], through establishing a model of obstructive jaundice-induced liver injury in mice, found that exercise combined with LRPS could improve the immune function of mice with liver damage. It reduced the expression of tumor necrosis factor-alpha (TNF-α), nuclear factor kappa B (NF-κB), transforming growth factor-beta (TGF-β), interleukin-6 (IL-6) mRNA in mouse serum, and restored liver cells in obstructive jaundice mice. In the study by Wang et al. [11], it was found that after ingesting LRPS, the compounds regulated cell membrane permeability, reduced oxidative stress in tissue cells, and scavenged free radicals in the body. This had a protective effect on rat liver damage, lowering the levels of malondialdehyde (MDA) and transaminases in rat serum, increasing the levels of glutathione peroxidase (GSH-Px), superoxide dismutase (SOD), and catalase (CAT) in the tissues. It was speculated that the protective mechanism of LRPS against liver damage was related to their antioxidant activity.

### 4.10. Prebiotic Activity

The gut microbiota constitutes a complex human ecological system, comprising over 1000 microbial species. It plays a crucial role in the host’s immune response, nutritional metabolism, and various life activities, maintaining a dynamic equilibrium with the host [68,69]. The results from the in vitro fermentation of LRPS indicate that gut microbiota can break down polysaccharides into short-chain fatty acids, resulting in a decrease in intestinal pH and lactic acid content. This suggests that LRPS have the ability to improve the intestinal environment [13]. Wang et al. [70] found that oral administration of LRPS increased the abundance of Lactobacillus and Bifidobacterium in the HFA mouse intestines. It decreased the levels of Enterobacter and Enterococcus, promoted the secretion of mucosal sIgA, and enhanced intestinal immune function in HFA mice.

## 5. Conclusions and Future Perspectives

*Lycium ruthenicum* Murr., as a dual-purpose medicinal and edible resource, has extensive applications in traditional ethnic medicine and food in China, and polysaccharide is one of its main active components. This review summarized the preparation methods, structural features, as well as the biological activities and their mechanisms of action in *Lycium ruthenicum* Murr. polysaccharides, and it was further clarified that *Lycium ruthenicum* Murr. polysaccharides had unique structural features and bioactive diversity. These characteristics provide broad prospects for their use in health foods, cosmetics, pharmaceuticals, and other fields. There has been considerable research on the primary structure, major biological activities, and mechanisms of action of LRPS, which is crucial for the subsequent development and application of these polysaccharides. However, current research suggests that LRPS may have even more diverse biological activities, and there is limited research on the advanced structures of these polysaccharides. This lack of knowledge about the relationship between advanced structures and biological activities hinders a comprehensive understanding. To more effectively develop and utilize LRPS, research could employ techniques such as X-ray diffraction, circular dichroism, and atomic force microscopy to study their advanced structures in the future. Building on this foundation, exploring the structure–activity relationship at the molecular level would enable the targeted development of functional health products or pharmaceuticals.

## Figures and Tables

**Table 1:** Study on the extraction of *Lycium ruthenicum* Murr. polysaccharides.

Number	Extraction Methods	Theory	Key Parameters	Advantages	Disadvantages	References
1	Hot water extraction	Polysaccharides are hydrophilic macromolecules, and water enters the cell through the cell wall, causing soluble components such as polysaccharides to dissolve out of the cell.	extraction temperature extraction time material ratio	Simple handling, polysaccharides are stable in hot water, it causes minimal damage to the polysaccharides.	Require high temperature and long extraction time.	[18,19]
2	Ultrasonic extraction	Under the action of ultrasound, high temperature and pressure appear inside the organism in order to accelerate the internal movement of molecules of substances, thus causing the deformation and rupture of the cell wall of living organisms, so that the effective components can be dissolved more quickly.	material ratio temperature ultrasonic power ultrasonic time	Simple and efficient, shorten the extraction time and increase the yield of polysaccharides	Ultrasound may disrupt the structure of the polysaccharides.	[20]
3	Microwave-assisted extraction	Through high-frequency microwave radiation, electromagnetic waves advance quickly into the cells, leading to the material internal absorption of the energy and thus rapid warming and instant pressurization, so that the cell wall breaks down, dissolving the required components.	material ratio microwave power temperature microwave time	Saves solvent, shortens extraction time, reduces energy consumption, and has a high yield.	Microwaves may disrupt the structure of the polysaccharides.	[21,22]
4	Ultrasonic–microwave synergistic extraction	Combine the dual action of ultrasound and microwave to further promote polysaccharides solubilization.	material ratio ultrasonic–microwave temperature power time	Time-saving, easy to operate, high yield	Ultrasound and microwaves may disrupt polysaccharide structure.	[23]
5	Enzyme-assisted extraction	Pectinase, papain and cellulase can disrupt the cell wall structure, reduce the mass transfer resistance of polysaccharide diffusion from intracellular to solvent, and increase the polysaccharide yield.	material ratio type of enzyme amount of enzyme digestion time digestion temperature pH	Rapid reaction, mild conditions, high yield; it is conducive to the protection of product activity, energy saving and environmental protection	It needs harsh conditions, incurs a high cost, and is rarely used alone, usually combined with other extraction methods.	[24,25]
6	High hydrostatic pressure assisted extraction	Under the action of ultra-high pressure, the internal and external pressures create a pressure difference inside the cell, promoting the rapid entry of solvents into the cell. When the pressure is released, it causes cell damage and rapid release of polysaccharides from the cell.	HHPE pressure HHPE time material ratio	Short time (2–10 min)	High pressure affects the dissolution of polysaccharides.	[20,26]
7	Dilute alkaline water extraction	Acidic polysaccharides or high molecular weight polysaccharides are generally more soluble in dilute alkaline solutions than in hot water.	extraction temperature extraction time material ratio	It is suitable for the extraction of some acidic polysaccharides, or high molecular weight polysaccharides	Extraction temperature should be kept below 10 °C, polysaccharides are degraded when the temperature is high.	[27]

## Data Availability

The original contributions presented in the study are included in the article, further inquiries can be directed to the corresponding author.
